# Aluminum-Filled Amorphous-PET, a Composite Showing Simultaneous Increase in Modulus and Impact Resistance

**DOI:** 10.3390/polym12092038

**Published:** 2020-09-08

**Authors:** Arfat Anis, Ahmed Yagoub Elnour, Mohammad Asif Alam, Saeed M. Al-Zahrani, Fayez AlFayez, Zahir Bashir

**Affiliations:** 1 SABIC Polymer Research Center (SPRC), Chemical Engineering Department, King Saud University, P.O. Box 800, Riyadh 11421, Saudi Arabia; aelnour@ksu.edu.sa (A.Y.E.); szahrani@ksu.edu.sa (S.M.A.-Z.); 2 Center of Excellence for Research in Engineering Materials (CEREM), King Saud University, P.O. Box 800, Riyadh 11421, Saudi Arabia; moalam@ksu.edu.sa; 3 SABIC Plastics Application Development Centre (SPADC), Riyadh Techno Valley, Prince Turki Street 1, P.O. Box 5101, Riyadh 11422, Saudi Arabia; fayezaf@sabic.com (F.A.); zbashir2703@gmail.com (Z.B.)

**Keywords:** polymer composites, metal-plastics, polymer metal composite, poly(ethylene terephthalate), PET

## Abstract

Metal-plastic composites have the potential to combine enhanced electrical and thermal conductivity with a lower density than a pure metal. The drawback has often been brittleness and low impact resistance caused by weak adhesion between the metal filler and the plastic. Based on our observation that aluminum foil sticks very strongly to poly(ethylene terephthalate) (PET) if it is used as a backing during compression moulding, this work set out to explore PET filled with a micro and a nano aluminum (Al) powder. In line with other composites using filler particles with low aspect-ratio, the tensile modulus increased somewhat with loading. However, unlike most particle composites, the strength did not decrease and most surprisingly, the Izod impact resistance increased, and in fact more than doubled with certain compositions. Thus, the Al particles acted as a toughening agent without decreasing the modulus and strength. This would be the first case where addition of a metal powder to a plastic increased the modulus and impact resistance simultaneously. The Al particles also acted as nucleating agents but it was not sufficient to make PET crystallize as fast as the injection moulding polyester, poly(butylene terephthalate) (PBT).

## 1. Introduction

Polymer composites filled with conductive particles are of interest for many fields of engineering. The thermal and electrical characteristics of such composites can be closer to metals, whereas the density and the article fabrication method are typical of plastics. Electrically conductive plastics are divided into two segments: enhanced but still low conductivity, for electrostatic-charge dissipating applications; and high conductivity for electromagnetic-interference shielding. Enhancement of both electrical and thermal conductivity is sometimes needed. In other instances, enhanced thermal conductivity with electrical insulation is desirable; for example, thermally conductive plastics are sought for heat dissipation in electronics-packaging applications, and because of the recent drive toward electric vehicles. Conductive plastics offer scope for all these variants.

Carbon black is a cheap additive for static charge dissipation but has limitations as high loadings are needed, which causes embrittlement. Graphite-PET composites have been reported by Alshammari et al. [1]. Although the electrical conductivity attained the minimum for electrostatic discharge applications (10^−6^ S/m) at a moderate loading of 14.7 wt. % of graphite, and the modulus increased by ~75%, the extension-to-break reduced to 1.5%. The last generally indicates brittleness [1]. Presently, newer carbon materials (carbon nanotubes or CNTs, graphene) are in vogue, for bringing conductive properties and enhancements in mechanical properties. These fillers have intrinsically high electrical and thermal conductivities, surpassing metals. However CNTs and graphene are currently too expensive [2]. For example, at current prices, CNTs are ~4200× and graphene ~3500× the price of aluminum powders. Even if the CNTs or graphenes are used with a loading of 1%, they are 50–70× more expensive than a plastic composite with 20% loading of Al. It is difficult to disperse the CNTs homogeneously within polymer materials, and some of the surface treatments used to improve adhesion between the CNTs with the polymer are cumbersome and simply impractical for scale-up [3,4]. The same applies to graphene composites [5,6]. A new trend is the study of hybrid conductive fillers such as CNTs or graphene, coupled with an inorganic material such as alumina, or carbon fibers, to tailor the thermal and electrical conductivity [7]. However, some of these hybrids involve even more difficult fabrication procedures.

Much work has been done in the past on metal powder filled conductive plastics [8]. Metal particles are cheaper and can be melt compounded with plastics readily. They yield composites with higher density than CNT composites, but can give electrical and thermal conductivity at a reasonable price, and therefore continue to be of practical interest for conductive plastics. Most commercially available electrically conducting plastics are based on metal powders and fine metal wires, while commercial thermally conductive plastics rely on alumina and boron nitride as fillers.

The various studies on metal-plastic composites show above a critical content of metal particles, a percolation threshold is reached above which the electrical conductivity shoots up by several orders of magnitude. The best case is if the percolation threshold is low, for example from 2–10 volume % (vol. %) loading. Sometimes percolation at low volume fractions is attained [9,10,11], but in other papers, higher metal loadings of 20–50% [12,13,14] are needed to achieve a leap in electrical conductivity. Particle shape is a factor that affects the percolation threshold. A low percolation threshold is possible when the metal particles are dendritic or wire-like. Jankovic et al. [9] made Cu-poly(methyl methacrylate) (PMMA) composites using copper powders with a highly dendritic structure. They obtained a very low percolation threshold of 2.9 vol. %. Likewise, Tekce et al. [15] showed the rise in thermal conductivity of Cu-polyamide 6 composites was higher when ribbon-like copper particles were used, instead of flakes and micro spheres.

Another interesting feature is that the percolation threshold can depend on the fabrication method. With some polymers, when compression moulding is used for sheet fabrication instead of extrusion or injection moulding, the percolation threshold for electrical conductivity is sometimes lowered. Dutta et al. [16] as well as Mamunya et al. [10] have shown that with certain polymers such as poly(vinyl chloride) (PVC), compression moulding of the polymer powder with metal powders such as Cu and Al creates a ‘segregated network’ which allows the percolation threshold for electrical conductivity to be reached at a low loading.

The mechanical properties of segregated network morphologies are likely to be poor, rendering the article unusable; this aspect is not considered in works like Dutta et al. [16] and Mamuniya et al. [10]).

Tavman and Evgin [17] described Al-filled high-density polyethylene (HDPE) for thermally conductive electronic-packaging applications. At the highest (50%) loading of Al, the thermal conductivity had risen about 5×. Generally, for thermal conductivity (unlike electrical conductivity), a very sharp rise does not occur till over ~70% loading. In their paper [17], the mechanical properties were not considered, but at 50% loading of Al, the composite was likely to be brittle. Indeed, many papers (Poblete et al. [18], Cu-PMMA; Pinto and Jiménez-Martín [19], Al flakes in nylon 6; Álvarez et al. [11], Al-PMMA) that reported a rise in electrical conductivity by 11–13 orders, focussed only on the electrical or thermal conductivity (or both), but neglected the mechanical properties. It is almost certain compositions such as Cu-PMMA would be weak and brittle. Nicodema and Nicolais [20] showed the adhesion of Fe and Al particles with styrene acrylonitrile (SAN) plastic was low and this resulted in a drop in mechanical properties over the base polymer (that is, despite achieving enhanced electrical conductivity, the mechanical properties were the drawback).

In papers where metal-polymer composites are made and mechanical properties are shown, it is generally observed that the tensile modulus increases modestly with filler content but often the tensile strength, the elongation-to-break, and the impact resistance, decrease compared with the base polymer, whether the article is made by compression moulding or injection moulding [13,14,21,22,23,24,25,26]. It is always difficult to get high stiffness with toughness. The decrease in impact resistance limits the use of filled plastic composites because in many engineering applications, toughness is a deciding factor in the final material selection.

There is only one work on metal-PET composites. Osorio-Ramos et al. [27] made Zn-PET composites using a compaction type process, but the mechanical properties were low. The past work on injection moulded metal-plastic composites have not selected PET for the matrix. However, there has been considerable work on injection moulding of clay-filled PET nano composites, driven by the desire to increase gas barrier for PET bottles. This trend has extended to injection moulding of PET composites with carbon nano tubes, graphenes and graphites [3,4,5,6,7].

This work is about Al-PET composites and their mechanical properties. Increases in thermal and electrical conductivities were not the primary focus here, but these should also accompany naturally. During vacuum metallization of biaxially oriented PET film, we observed the deposited aluminum adheres strongly to the PET, while with PP and PE films, the surface has to be treated with a corona, otherwise the aluminum can be wiped off. Hooper et al. [28] showed that vapour deposited aluminum films can form organometallic bonds with substrates containing C=O groups (as present in polyesters). We had observed also during compression moulding PET between two aluminum foils (used as peelable backing to give clean films), the foils could not be peeled off from the PET sheet due to very strong bonding between the two; in contrast, Al backing foils can be peeled off quite easily from sheets of polyethylene or polypropylene that had been compression moulded between them. Aluminum generally forms an oxide layer that is 6 nm thick, hence unlike the vacuum metallization, in the case of aluminum foil bonding to PET, the adhesion may be due to interaction between its oxide layer and the PET melt, rather than due to organometallic bonding. Either way, we thought that there was the intriguing possibility that ductile aluminum powder particles might adhere to the PET (even with the oxide coating), and might act as an impact toughener, although in almost all recorded cases, large loadings of any rigid filler decreases the impact resistance.

## 2. Materials and Methods

### 2.1. Materials Used

PET (BC 212 grade, SABIC, Riyadh, Saudi Arabia) with I.V. of 0.84 dL/g was selected for the preparation of the composites. It was in the form of pellets.

Two aluminum (Al 1 and Al 2) powders from Nanokar, Turkey, were tried. The particle size characteristics and other properties of the aluminum powders as per the manufacturer’s data sheet are shown in Table 1. The powders were grey in appearance.

### 2.2. Melt Extrusion Compounding

Composites of PET and aluminum were prepared using a DSM Xplore micro-compounder (Geleen, The Netherlands). It had a co-rotating twin screw extruder and was provided with a melt re-circulation system for repeated mixing. Before extrusion, the PET pellets were dried overnight in a Hereaeus fan oven at 150 °C. The filler content of the micro-Al l/PET composites was varied between (5, 10, 15, 20, 25 and 30 vol. %), whereas nano-Al 2/PET composites were prepared with aluminum loadings of (1, 3 and 5 vol. %). To obtain a homogeneous dispersion of aluminum particles in the PET matrix, the melt compounding temperature and the screw speed were set at 275 °C and 100 revolutions per minute, respectively. The residence time in the extruder was measured as the time interval between the time when the mixture entered the entrance of extruder barrel and the time the extrudate came out of the die. The residence time was kept around 3~5 min.

### 2.3. Injection Moulding of Al-Compounded PET

For the preparation of the standard test specimens for the characterization of the composites, the compounded melt was injection moulded in a DSM Xplore Micro-injection moulder (Geleen, The Netherlands). The DSM Xplore micro-compounder (15 cm^3^) allowed the molten mix to be collected in a heated vessel which could be connected to the micro injection moulding unit (12 cm^3^). The injection moulding parameters for preparing the amorphous PET bars were set as follows:

- Melt temperature of (275 °C)
- Mould temperature (~25 °C).
- Injection pressure of (6 bar).
- Injection time of (45 s).

The injection moulded articles were (1) tensile bars (2) flexural bars (3) Izod impact bars and (4) square plaques. The dimensions were (148 mm × 12.70 mm × 3.25 mm) for the standard tensile bars; (134 mm × 12.80 mm × 3.20 mm) for the flexural bars, (64 mm × 12.80 mm × 3.20 mm) for the Izod impact bars and (40 mm × 40 mm × 2.0 mm) for the square plaques used in the thermal conductivity measurements. Note that blanks (i.e., pure PET) were also injection moulded. These were uniformly transparent suggesting they were amorphous. The Al-PET articles were opaque and grey in colour.

The composites’ formulations (based on volume percentage), the corresponding weight percentages, and the calculated densities of the micro-Al 1/amorphous PET and nano-Al 2/amorphous PET composites are shown in Table 2 and Table 3 respectively. The weight of Al powder needed for the selected volume % was calculated from the density of Al (2.71 g/cm^3^). The composites’ theoretical densities *ρ* were calculated according to Equation (1), which uses the density values of the individual constituents and the weight percentage of each constituent.

(1) $$\rho =\frac{1}{\sum_{i=1}^{n}\frac{w_{i}}{\rho_{i}}}$$

where: *w*_i_ is the weight fraction (=weight percentage/100) of each constituent and *ρ*_i_ is the density value of the individual constituents (*ρ*_Al_ = 2.71 g/cm^3^ and (*ρ*_PET_ = 1.333 g/cm^3^ for amorphous PET).

### 2.4. X-ray Diffraction

The Wide Angle X-ray diffraction analysis was performed at room temperature on the Al powders and the injection moulded amorphous PET and the Al-PET composites, using a Bruker (D8 Discover, Karlsruhe, Germany) diffractometer operating at 40 kV and 40 mA, to produce Cu Kα radiation. The scanning speed was 2°/min and the range was 2θ = 10°–80°.

### 2.5. Scanning Electron Microscopy (SEM)

Both Al 1 and Al 2 powders were examined with a scanning electron microscope (JSM-6360A, JEOL Ltd., Akishima, Japan) to see their particle shape and confirm the particle size reported in the manufacturer’s data sheet.

The fractured surfaces of the amorphous PET and the Al/PET composite samples were also examined in the SEM. All samples were sputtered with a thin layer of gold and mounted on aluminum holders by using double-sided electrically conducting carbon adhesive tape.

### 2.6. Thermal Analysis

Differential Scanning Calorimetric (DSC) studies of the Al/PET composites were carried out using a DSC-60A (Shimadzu, Tokyo, Japan) Thermal Analyzer, according to the ASTM D-3418 standard testing procedure. The amorphous PET and the Al/PET composites were heated from 30 to 300 °C at 10 °C/min, held at the maximum temperature for 3 min, and then cooled to the minimum temperature (30 °C) at 10 °C/min.

### 2.7. Mechanical Analysis

#### 2.7.1. Tensile Testing

The tensile properties of the Al/amorphous PET bars and amorphous PET bars were characterized with a Tinius Olsen uniaxial universal testing machine (Horsham, PA, USA, Model: H100KS), according to the ASTM D-638 standard testing procedure. The standard tensile bars of dimensions (148 mm × 12.7 mm × 3.25 mm) were properly fitted to the machine grips, and the tests were carried out at room temperature (cross head speed of 50 mm/min). Each test was repeated at least five times per composite sample to ensure repeatability, and average values with standard deviations are reported. The stress-strain curves were recorded online and tensile strength, Young’s modulus and elongation-at-break of the composites were obtained from the stress-strain data.

#### 2.7.2. Flexural Testing

The flexural properties of the Al/amorphous PET composites and the amorphous PET bars were investigated by using the Tinius Olsen uniaxial universal testing machine, according to the ASTM D-790 standard testing procedure. The test was carried out at room temperature through a three-point bending loading system with support span length of 50.8 mm and at a crosshead speed of (10 mm/min). Each test was repeated at least five times for each composite sample to ensure repeatability, and average values with standard deviations are reported. The force-extension curves were recorded online and both the flexural strength and flexural modulus of the composites were calculated accordingly.

#### 2.7.3. Notched Izod Impact Resistance

The notched Izod impact resistances of the Al/amorphous PET composites and the amorphous PET were measured using an AMSE Multi Impact Tester (Lombardy, Italy), according to the ASTM D-256 standard testing procedure. The Izod bar was notched with a notch depth set at 2.5 mm with an angle of 45° and a reaming depth under the notch of 10.3 mm. The tests were carried out at room temperature with a pendulum energy of 5.5 J, a rising angle of 118° and an impact speed of 3.50 m/s. Each test was repeated at least ten times for each composite sample to ensure repeatability.

### 2.8. Thermal Conductivity Measurement

The thermal conductivity of the square plaques samples (40 mm × 40 mm × 2.0 mm) was measured in triplicate using a TCi Thermal Conductivity Analyzer from C-Therm Technologies (Fredericton, NB, Canada) using a Modified Transient Plane Source Sensor conforming to ASTM D7984. The instrument uses a single-sided, interfacial heat reflectance sensor that applies a transient, constant heat pulse of 1 to 3 s to the sample, and the design is such that the heat transfer is one dimensional through the thickness of the sample.

## 3. Results and Discussion

### 3.1. Aluminum Powder-Characterization

**Figure 1:**
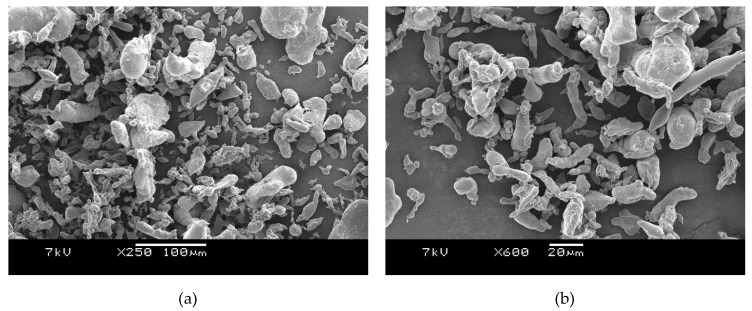
SEM images of (**a**) Al 1 micro-powders (250× Magnification) showed bigger particle sizes than in the manufacturer’s data sheet in (**b**) Al 2, nominally nano powders (600× Magnification) also showed much bigger particle sizes than in the manufacturer’s data sheet.

Aluminum powders are available with different particle shapes: spherical, flake and irregular. The as-received aluminum powder samples from Nanokar where characterized through SEM in order to confirm their particle shape and size. The SEM images of the micro and nano powders are shown in Figure 1a,b. Both the aluminum samples here had irregular particle shapes. The particle size of the micro powder Al 1 as reported by the manufacturer was 3 µm (average), while that of aluminum nano powder Al 2 was between 40–60 nm. However, the particle sizes of both these samples in the SEM images were much higher than the values in the manufacturer’s data sheet. The SEM picture of the micro powder in Figure 1a shows particles of 70–80 µm while the supposed nano powder in Figure 1b had some carrot-like particles with lengths of up to 20–60 µm. It is not clear whether this was due to agglomeration or the manufacturer’s data sheet claiming a wrong particle size. We can only refer to Al 2 as nominally a nano powder. In effect, we have two micro particle powders, with Al 1 having a larger size than the Al 2. The Al 2 powder was a darker grey which is indicative of finer particles.

**Figure 2:**
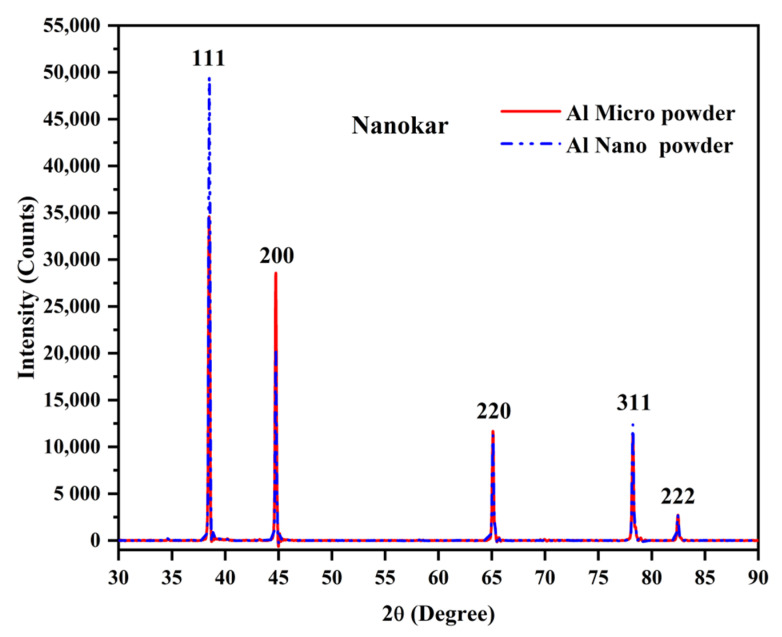
X-ray diffractograms of the Aluminum powders.

The X-ray diffractograms of the two Al powders recorded with Cu Kα radiation are presented in Figure 2. This shows the typical pattern of pure aluminum with sharp peaks at 2θ values of (38.415, 44.66, 65.041, 78.150, 82.335°) corresponding to Miller indices of (111, 200, 220, 311 and 222), respectively. Thus, there was no issue with the purity of the Al cited in the manufacturer’s data sheet; there would be an oxide layer but this cannot be detected.

Pure aluminum has a modulus of 70 GPa and is a malleable and ductile metal, with a strength of ~90 MPa and an extension-to-break of 50–70%. It is a good conductor of heat (with a thermal conductivity of 204 W/m K) and electricity (with an electrical conductivity of 3.538 × 10^5^ S/cm), it has a low density (for a metal) of 2.71 g/cm^3^, and a melting point of 660 °C. By alloying with other elements, the modulus does not change much, but the strength of the aluminum can be increased to 690 MPa. Here, a pure Al was used.

### 3.2. X-ray Diffractograms of the Composites

**Figure 3:**
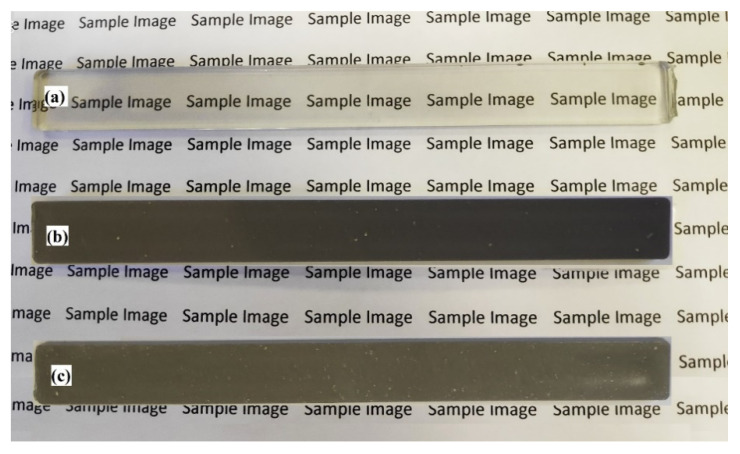
Appearance of the (**a**) Amorphous PET, (**b**) Al-PET composite with nano Al 2 and (**c**) Al-PET composite with micro Al 1. Note that the bar with micro Al shows speckle due to large specks of shiny Al particles.

**Figure 4:**
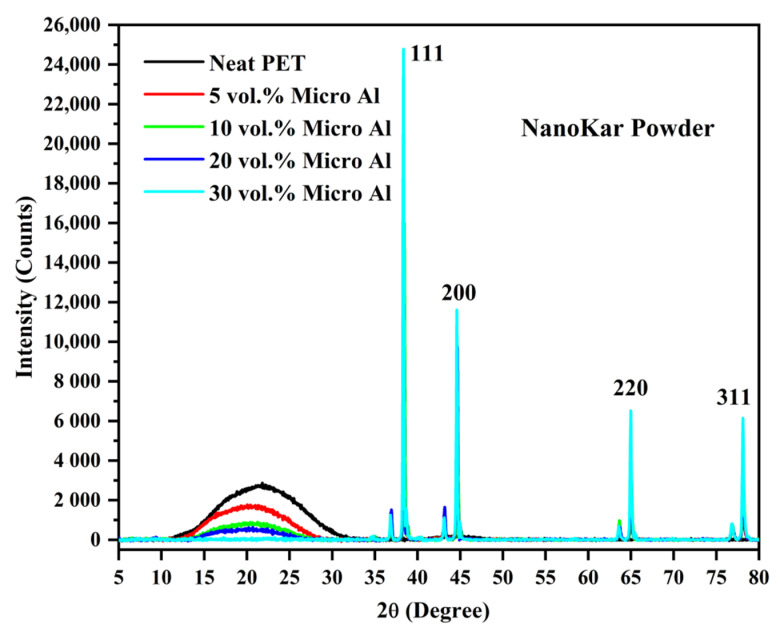
X-ray Diffractograms of the micro-Al 1/amorphous PET composites. The broad peak at 2θ values between 11° and 33° confims the PET is in an amorphous state.

**Figure 5:**
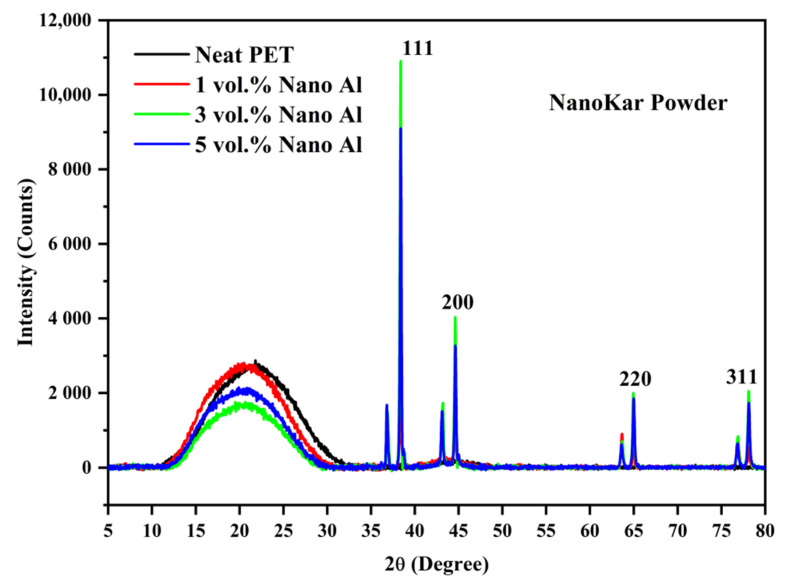
X-ray Diffractograms of the nano-Al 2/amorphous PET composites. The broad peak at 2θ values between 11° and 33° confims the PET is in an amorphous state.

The pure PET bar moulded with cold moulds was transparent (Figure 3). Generally, in un-oriented PET, the transparency is associated with its amorphous state. If the un-oriented PET bars had crystallized, they would appear white. The Al-PET bars moulded with cold moulds were opaque and grey (see Figure 3) and from visual inspection, it was not obvious if the PET in the composites was also in an amorphous state. The composite from the micro Al 1 in Figure 3 (Bottom) showed speckle. These are shiny specks arising from particles that can be seen by the naked eye (that is about 0.2 mm and above). The composite from the nano Al 2 powder was a darker grey with less speckle. The literature on other metal-polymer pairs had shown that the metal particles can act as crystallization nucleators for the polymer (Rusu and Rusu [23], Cu-PA6)). However, the X-ray diffractograms of the Al/PET composites (moulded bars from Al 1 and Al 2) in Figure 4 and Figure 5 showed a broad peak at 2θ values between 11° and 33°, indicating the PET portion was in the amorphous phase. The intensity of this broad amorphous PET peak decreased with increased filler loading due to the decreased content of the PET phase. Also in Figure 4 and Figure 5, the typical sharp peaks of aluminum powders can be seen at 2θ° (38.415°, 44.66°, 65.041°, 78.150°, 82.335°) with a relatively larger intensity compared with the amorphous PET peak.

### 3.3. Morphology and Thermal Analysis of Amorphous PET with Micro-Al 1 and Nano-Al 2

**Figure 6:**
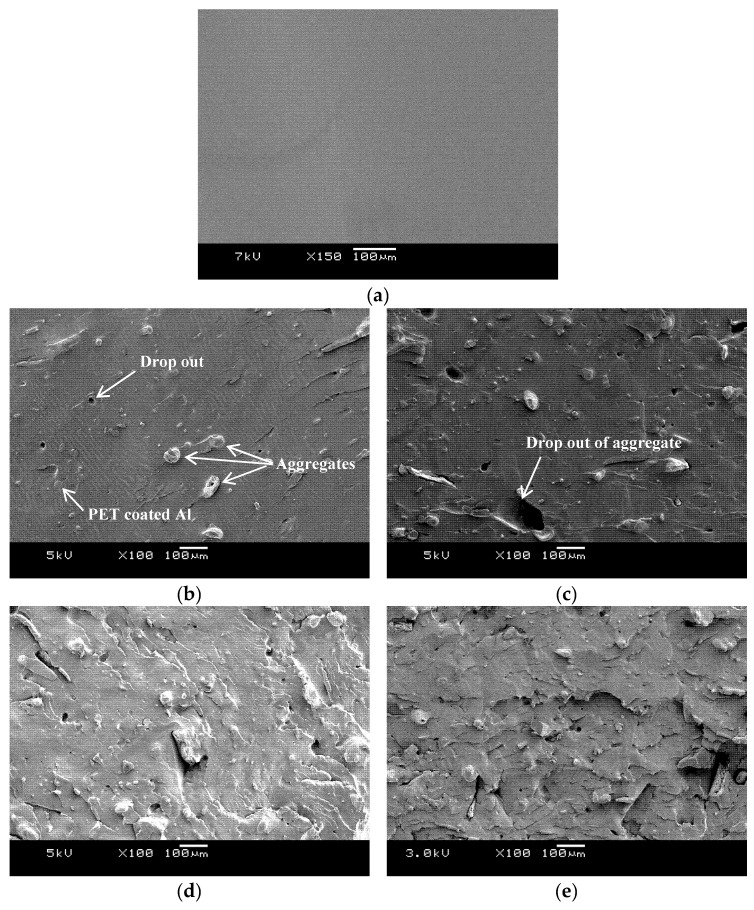
SEM images of (**a**) Neat PET is featureless. Micro-Al 1/PET composites (**b**) 5 vol. % Al (**c**) 10 vol. % Al (**d**) 15 vol. % Al (**e**) 20 vol. % Al.

The fracture surface-morphology of the amorphous PET bar and the micro-Al 1/amorphous PET composites at different loadings of aluminum micro particles is shown in Figure 6. The pure PET in Figure 6a is featureless at this magnification. Figure 6b with 5 vol. % shows the finer Al 1 particles are embedded under a coating of PET, but larger 100 μm particle/aggregates, protrude. Figure 6c, with 10 vol. % of Al 1, shows an area where a large aggregate (even greater than 100 μm) has fallen out. Figure 6d with 15 vol. % shows most of the particles are coated with PET with few drop outs. Figure 6e shows the deformation pattern tended to change from smooth to brittle, as the aluminum loading increased beyond 15% to 20%. There are a few voids in the fracture surface due to the drop-out of Al particles. Since the number of voids was much smaller compared with the number of the well embedded Al particles, this indicates that the interfacial interaction between the Al particles and the PET matrix was good overall.

**Figure 7:**
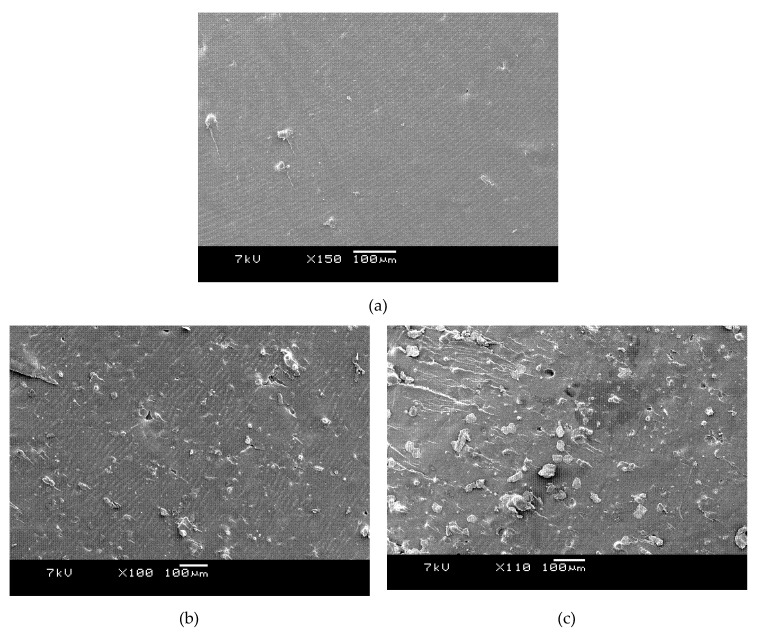
SEM images of nano-Al 2/PET composites (**a**) 1 vol. % Al 2 (**b**) 3 vol. % Al 2 (**c**) 5 vol. % Al 2. There are a lot of fine particles but agglomerates as large as 50 µm can be seen. There are very few drop-outs (dark cavities).

The fracture surface morphology of the nano-Al 2/PET composites at different loading percentages of the aluminum nano particles is shown in Figure 7. Comparing Figure 7b (3 vol. % Al 2) with Figure 6b (5 vol. % Al 1), it can be seen the former has more particles than the latter as would be expected with finer particle division. However, although the manufacturer’s data sheet claimed particle sizes in the range of 40–60 nm the size (Table 1), some big particles (50 µm) are also seen in Figure 7, which indicates the formation of agglomerates that were not disintegrated by the shearing in the twin screw extruder during melt blending.

**Figure 8:**
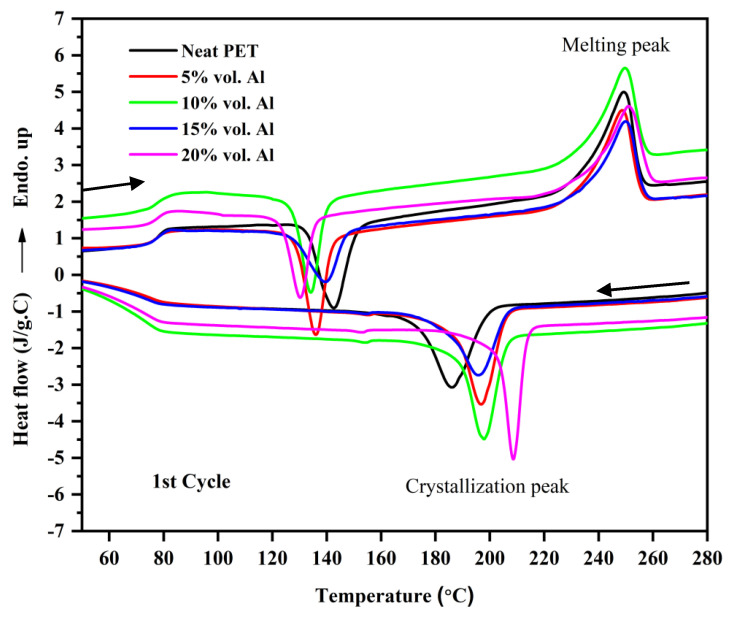
Thermograms of the first DSC heating-cooling cycle of amorphous PET and micro-Al 1/amorphous PET composites. The top curves show heating to the melt; the bottom shows cooling curves from the melt.

The thermal behavior of the first DSC cycle of neat PET and micro-Al 1/PET composites is shown in Figure 8 and the numerical data obtained from their corresponding DSC thermograms are summarised in Table 4. In Figure 8, first at ~80 °C, a strong glass transition is seen in the form of a sigmoidal change in the baseline, which is indicative that the PETs in all the moulded samples were amorphous. Note, that amorphous PET (indeed any amorphous material) can show ‘physical ageing’ after a period of storage, which leads to a decrease in free volume and embrittlement [29]. In the DSC curve of a physically aged polymer, at the *T*_g_, there would be hook or peak at the start of the sigmoidal change in the baseline, and this increases with storage time [29]. This is not seen in any of the heating curves in Figure 8 and this indicates that significant ageing had not taken place, and hence embrittlement due to physical ageing would not cloud the results and conclusions of the impact tests (to be discussed later). After the *T*_g_, a typical cold crystallization peak in the range (130 to 143 °C) for the PET and micro Al/PET composites can be seen. The existence of a cold crystallization peak also correlates with the PET being in an amorphous or low crystallinity state; such a peak would not be observed in the first heating scan had the PET bar been highly crystalline. The X-ray diffractograms in Figure 4 had confirmed the PET was amorphous in both the pure material and in the composite. Regardless of the Al particle content in the PET matrix, both the glass transition temperature (*T*_g_) and melting temperature (*T*_m_) of the micro-Al 1/PET composites remained nearly the same as compared with the amorphous PET. However, the cold crystallization temperature (*T*_cc_) of the micro-Al 1/amorphous PET composites was significantly altered (shifted to lower temperature) with the incorporation of Al. Also from Table 4 and Figure 8, it can be seen that the *T*_c_ (the peak crystallisation temperature from the melt) shifted towards higher temperatures and the shift widened with increased Al loading. The neat PET sample showed a *T*_c_ of 186 °C, while that of the 20 vol. % Al 1 loading was around 209 °C.

The shifts seen in the *T*_cc_ and the *T*_c_ (Table 4 and Figure 8) indicate that the aluminum particles impart a good nucleating effect. Rusu and Rusu G [23] had found a similar nucleating effect for crystallization in nylon 6 (PA6) filled with copper (Cu) powder. However, the nucleating effect was still not good enough to make the 3.2 mm thick PET bars crystallize when using a cold mould as shown by the X-ray in Figure 4. Pure poly(butylene terephthalate) (PBT) on the other hand would crystallize even if a cold mould is used.

Clays, graphenes and CNTs are reported as nucleating agents for PET. Shabafrooz et al. [5] melt compounded 2–10% by wt. of graphene with PET using the same micro-compounder and micro-injection moulder as used here. Their injection moulded PET bars with graphene were also amorphous and the first heat showed the cold crystallisation occurred at lower temperatures in the samples with graphene compared with the pure PET, while for crystallization from the melt, the composites with graphene crystallized at a higher temperature. That is, the same trend was observed as in Figure 8 here with the PET containing aluminum. However, in their work the crystallization of the PET was faster as the exothermic peak on cooling from the melt was at ~200 °C while it is at ~185 °C for the PET here in Figure 8. This difference is because Shabafrooz et al. [5] used a lower molecular weight PET (0.61 dL/g) than us (0.84 dL/g), and generally the PET with lower I.V. crystallizes faster than PET with higher I.V. Aoyama et al. [6] made a comparison of CNTs versus graphene on the crystallization of PET from the melt; both showed a nucleating effect, but graphene had a stronger effect. Graphite in PET composites also shows a nucleating effect [1]. As with Al here, in all these works [1,5,6], the fillers acted as nucleating agents, but they did not make the PET crystallize so fast that the moulding was semi-crystalline instead of amorphous.

If a crystallizable polymer crystallizes fast, it can be used to make crystalline articles via injection moulding no matter what the thickness; an example is polyethylene. In some cases, a crystallizable polymer crystallizes so slowly that it gives uniformly amorphous articles, no matter what the thickness; an example is polycarbonate. PET is rarely used as an injection moulding thermoplastic for end-use articles. The reason for this is that PET has an intermediate crystallization speed and hence it is possible to get articles in various states from amorphous to semi-crystalline, depending on the part’s thickness. In a thin-walled injection moulded article, the PET will be amorphous and transparent if cold moulds are used. If the part is thick walled, PET will give on injection moulding a skin-core effect (skin is transparent and amorphous, core is crystalline and white, see ref. [30] for a picture). It is difficult to make a uniformly crystallized, thick PET part by injection moulding, unless hot moulds are used. Hence, for most injection moulding applications where uniformly crystallized parts are needed, poly(butylene terephthalate) (PBT) polyester is used instead of PET. The crystallization rate depends on crystal nucleation as well as crystal growth rate, and the latter is intrinsically slower in PET compared with PBT as the PET chain is more inflexible than PBT.

**Figure 9:**
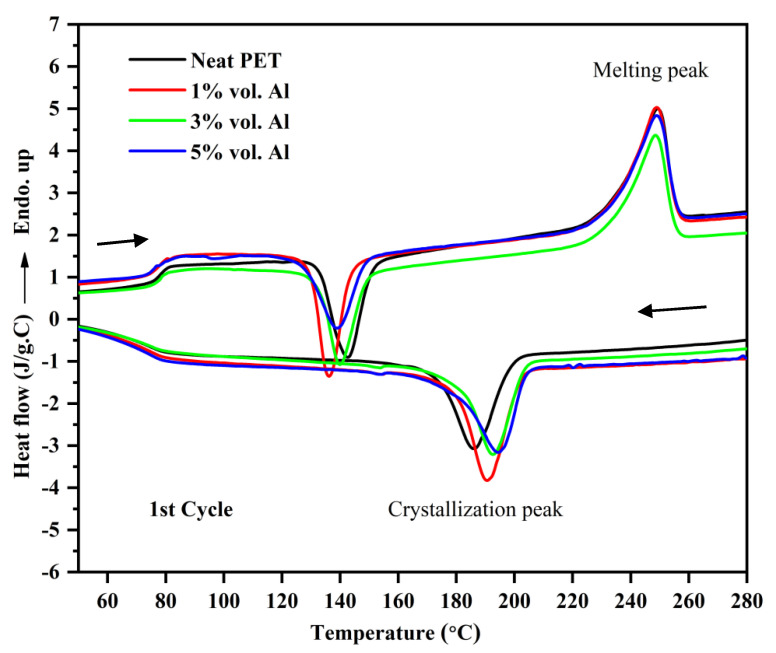
Thermograms of the first DSC heating-cooling cycle of PET and nano-Al 2/PET composites.

The thermal behavior of the first DSC cycle of the nano-Al 2/amorphous PET composites is shown Figure 9. Similar to the thermograms of micro-Al 1/amorphous PET composites, regardless of the loading of the Al 2 particles, both glass transition temperature (*T*_g_) and melting temperature (*T*_m_) of Al 2/PET nano-composites remained virtually unchanged. Again, there is no peak or hook at the *T*_g_ in the melting curves in Figure 9, which means the amorphous PETs (with and without Al) had not physically aged. The typical cold crystallization peak for the neat PET and the Al 2/PET nano-composites also indicates the PET in all the samples was in the amorphous phase. The cold crystallization temperature *T*_cc_ shifted toward lower temperatures and the degree of shift widened with increased Al 2 loading. However, the shifts in the *T*_cc_ of the nano Al 2 /PET composites were significantly lower (up to 4.7 °C relative to pure PET, Table 5) than those of the micro Al 1/PET composites (up to 12.6 °C, see Table 4). For the shift in *T*_c_ (the peak crystallization temperature while cooling the melt), in the nano Al 2-PET composite, it was up to 11.9 °C relative to pure PET melt (see Table 5), while it was up to 22.6 °C with the micro Al 1 composite relative to the pure PET (see the 20% composition Table 4).

### 3.4. Mechanical Properties of Al-Amorphous PET Composites

#### 3.4.1. PET Blended with Al 1 Particles

Table 6 shows the tensile and flexural properties of the amorphous PET composites with the Al 1 particles. The tensile modulus increased continuously with increased Al loading. The value of tensile modulus for amorphous PET was 1.60 GPa, while that of the Al 1/PET composites filled with 15 vol. % Al micro particles reached 2.07 GPa. The tensile strength remained invariant at ~60 MPa at all loadings of micro Al. That is, unlike most cases in the literature, there was no major drop in the tensile strength with filler content. Osman and Mariatti [24] showed that in polypropylene filled with Al particles, the tensile strength dropped monotonically; by 30% vol. of Al, the tensile strength of the Al-PP composite had halved. Similar drops in strength are reported in other metal-plastic pairs [20].

Table 6 shows the elongation-to-break of amorphous PET at room temperature (with a cross head speed of 50 mm/min) was 96%. The elongation-to-break increased to 136% at 5 vol. % of Al 1 but the variation was high. At higher loadings of Al 1 like 10 vol. % and 15 vol. %, the elongation decreased abruptly to 13–16%. The abrupt drop in elongation-to-break is commonly observed in filler composites. Surprisingly, the notched Izod impact resistance increased at these concentrations despite the decrease in elongation-to-break, as will be discussed shortly.

Table 6 shows also the flexural modulus values increased as the Al 1 content increased. Al 1/PET composites filled with 15 vol. % Al 1 particles showed the highest value of flexural modulus (3.24 GPa), which was about 24% higher than the flexural modulus of the amorphous PET (2.47 GPa). The incorporation of the Al 1 particles into the PET matrix led to no statistically significant change in the flexural strength of the Al 1/PET-composites; it was about 90 MPa. Tensile bars with 20 vol. % and 30 vol. % of Al 1 could not be moulded without defects, hence they were not usable for the tensile and flexural tests. Thus, Table 6 shows the tensile properties only up to an Al 1 content of 15 vol. %. Note with Izod bars, we could mould with up to 30 vol. % of Al 1.

Table 7 shows the notched Izod impact resistance of the amorphous PET and the composites made with Al 1 particles. Note that in the literature, this is often called ‘notched Izod impact strength’, but we prefer to call it a ‘notched Izod impact resistance’. Strength is based on a force, whereas the Izod impact involves an energy and the value is given in J/m or J/m^2^. Table 7 shows that the addition of Al 1 to the amorphous PET samples resulted in a significant enhancement of the notched Izod impact resistance. However, the standard deviations in Table 7 are higher than for the tensile and flexural tests (see Table 6), hence some care has to be taken in the interpretation.

In notch-sensitive materials, the impact results tend to show more variation than the tensile modulus. Ideally, if impact toughening occurs, the magnitude of the effect should be large enough to be meaningful and the measurement should be precise enough to be trustable. Hence, a two sample (or unpaired) *t*-test for the Izod impact resistance values for the micro-Al composites was conducted, to see if the mean values of impact resistance in Table 7 for two compared compositions of Al-PET were significantly different (see Table 8). The two sample *t*-test determines whether or not the means of two independent populations from two normal distributions are equal or whether they differ according to a significance level. We make the assumption the Izod test results are normally distributed for each composition (population) and while their true mean values may differ, their true standard deviations are the same. For the unpaired *t*-test, we used the null hypothesis H_null_: μ_Al x%_ − μ_Al y%_ = 0, that is, there is no difference in the means, and the alternative hypothesis applied was H_a_: μ_Al x%_ − μ_Al y%_ ≠ 0, where μ is the mean Izod impact values for compositions with x% Al and y% Al. A significance level of 0.05 was chosen for the comparison of the means. This indicates that there is a 5% risk of rejecting the null hypothesis when it is true (or there is a 5% chance of wrongly concluding that there is a difference between Izod impact values of x% Al and y% Al, when there is no actual difference between the two populations).

Inspection of Table 7 shows that the impact resistances of all the Al 1–amorphous PET compositions are higher than the amorphous PET; that is, the Al 1 particles act as a toughening agent for amorphous PET. This is reflected in the minimum, median and maximum values also, which are higher than the corresponding values for the amorphous PET. Due to the higher variation in impact results, the *t*-test analysis in Table 8 must also be considered for interpreting the results. The general conclusion from Table 7 and Table 8 is (1) the impact strength increases up to 15 vol. % of Al 1, reaching a maximum of 51.6 J/m which is more than double the value of amorphous PET (2) between 15 and 20 vol. %, there is a levelling or no real difference (3) the 30 vol. % shows a decline versus the 15 vol. %, but its impact value does not fall below the level of the 10 vol. % Al 1. The SEM fracture surfaces in Figure 6d,e suggest there is a transition to a more brittle fracture after 15 vol. % of Al 1. The standard deviations in Table 7 for 20 and 30 vol. % have become very large and in doing the *t*-test, there is the assumption that the two normal distributions for each population are the same or close (the ratio of the higher to the lower standard deviation should be at least < 2 for applicability of the test). The higher variation in Table 7 after 15 vol. % is due to the higher tendency for agglomeration. However, even at high loadings, the values of the 20 vol. % and 30 vol. % Al 1-amorphous PET do not decrease below the base amorphous PET. Thus, we can say with a high degree of confidence that Al 1 particles cause impact toughening of amorphous PET.

Even with the poor quality of the powder, it is remarkable that unlike most cases in the literature, where addition of metal particles decreased the impact resistance [20], with amorphous PET, the impact resistance more than doubled when Al was added. Taşdemır and Gülsoy [31] showed that in HDPE, polypropylene, and polystyrene filled with 5, 10 and 15 vol. % iron powder, the notched Izod impact resistance decreased in all three cases, and there was a 50% reduction with HDPE containing 10 vol. % of Fe.

#### 3.4.2. PET Blended with Al 2 Powder

The nano Al 2 powder was incorporated up to only 5 vol. %. Table 9 shows the tensile modulus and tensile strength of the Al 2/amorphous PET composites were almost invariant (~1.60 GPa for tensile modulus, and ~56 MPa for tensile strength) with the volume percentage (of Al 2 particles. There is not much literature on the mechanical properties of metal-filled PET, so the best we are able to compare is with graphene-amorphous PET composites from recent work. Shabafrooz et al. [5] reached a tensile modulus of ~1.8 GPa with 2 wt. % of graphene in amorphous PET and this could be raised to 2.4 GPa with graphene that had been surface-modified with trimellitic anhydride. The level of modulus attained is not very different from the Al-PETs in Table 6, but due to the current cost of graphene (even with 2% loading), and the surface treatment procedure needed [5] to create bonding to the PET, the graphene-PET composite would be less worthwhile than the Al-PET.

**Figure 10:**
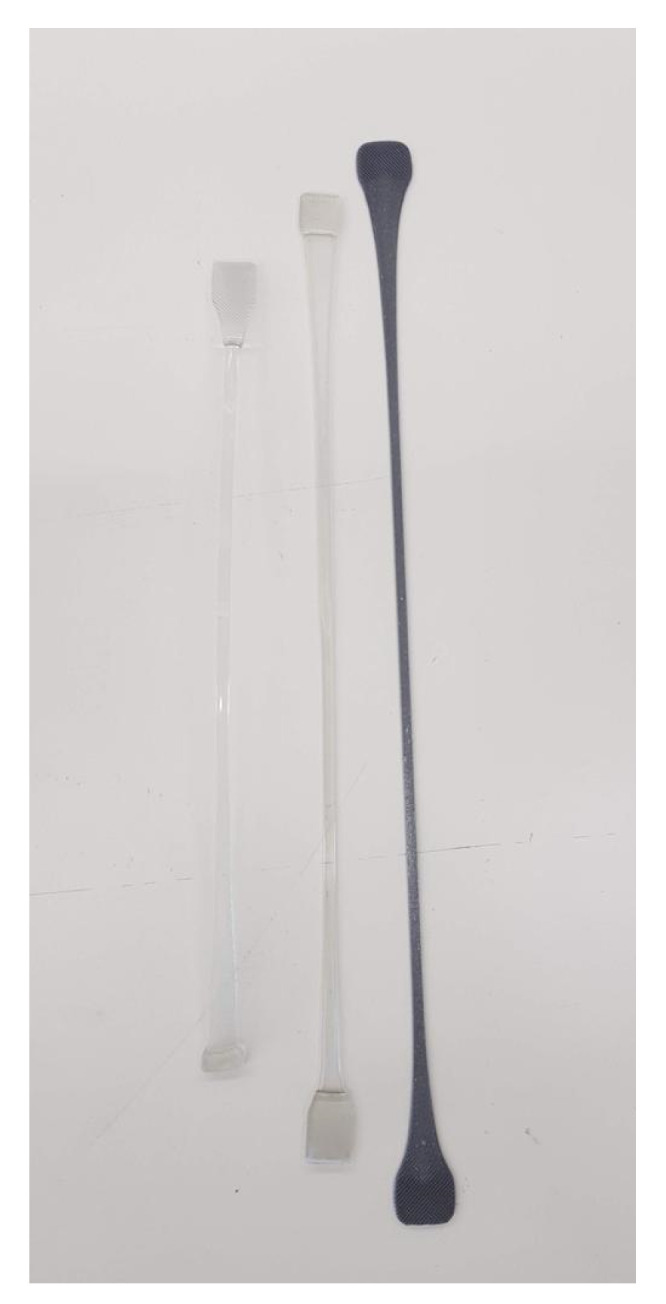
Injection moulded tensile bars were hot drawn ~5× at 100 °C and heat set at 170 °C under tension. The two transparent samples are amorphous PET after drawing to two different draw ratios, and crystallizing under tension. The grey sample is a 3 vol. % Al 2-PET, also drawn and crystallized under tension.

The flexural moduli and strengths of the nano-Al 2/amorphous PET composites were also invariant with the Al content in the range studied. However, the elongation-to-break somewhat surprisingly showed an increase at 1 and 3 vol. % (Table 9). It is well known that amorphous PET is easily drawn 5–6× *above its T_g_*, typically at 90–120 °C, by a necking process; it is the basis of PET fibre and filament production, and the production speed is well over 1000 m/min. Less well known is that amorphous PET can neck and draw even at room temperature to 500% if low cross head speeds, such as 2 mm/min, are used. In the current case, the amorphous PET extended up to 96% (average value with high standard deviation) at room temperature at the cross head speed used in the tensile test (50 mm/min). Surprisingly, the 1 vol. % and 3 vol. % Al 2-amorphous PET tensile bars actually extended over 400% at room temperature at 50 mm/min, and the variation was low (see standard deviations in Table 9). At 5 vol. % Al 2, there was a drop in extensibility, with increased variability compared with the 1 and 3 vol. % loading. Figure 10 shows a tensile bar of a 3 vol. % Al 2-PET drawn to high draw ratios of ~5:1 at 100 °C and heat set at 170 °C under tension. Two unfilled PET bars, drawn at 100 °C and heat set at 170 °C under tension, are also shown. Note that drawn bars of pure PET are transparent (Figure 10), although after drawing and heat setting, they transform from amorphous to semi-crystalline. Amorphous PET will be transparent always; semi-crystalline PET will be opaque if thermally crystallized but transparent if formed by stretching of amorphous PET (for example PET bottles are transparent). The unimpaired drawability of compositions of amorphous PET with up to 3 vol. % of the nano Al 2 would be suitable for making Al-PET filaments by hot stretching. In most of the other studies on metal-polymer composites, the oriented form is not considered, but as PET is the principal synthetic fibre of the textile industry, this was useful to establish, as conductive filaments, or fabrics with reduced static build up may be possible with Al. PET with genuinely nano-sized Al should be melt-spinnable without blockage of the spinnerets, and the filaments would be hot drawable like pure PET, up to some percentage of Al.

The notched Izod impact resistances of the Al 2/amorphous PET composites are listed in Table 10. As with the Al 1/PET composites, the addition of nano aluminum particles produced a major enhancement in the composites’ impact resistance. With the Al 2, an impact resistance of 51 J/m was achieved with 5 vol. % Al and this is more than double the impact value of the amorphous pure PET.

Table 11 uses the *t*-test to analyse the mean values of notched Izod for the amorphous PETs with Al 2 particles. All compositions showed an increase in impact resistance compared with amorphous PET, and with 5% Al 2, the impact resistance was more than double of amorphous PET. The standard deviation of the 5% composition was about half that of amorphous PET (Table 10), hence even without the *t*-test, the increase is obvious. Compositions 1% and 3 vol. % of Al 2 were not statistically different from each other. Often with nano particles, effects found at higher loadings with micron sized particles can be replicated with lower loadings if agglomeration does not take place; this is seen here with the notched Izod impact. Doubling of the impact resistance occurred with micron sized Al 1 at 15 vol. % but this effect was seen at 5% with Al 2 (compare Table 10 and Table 11 with Table 7 and Table 8). However, this was surprising since the SEM micrographs had indicated the presence of some micron sized Al particles in the starting nano Al 2 powder (see Figure 1b). Perhaps after melt mixing, some of the micron-sized aggregates in the nano Al 2 powder were broken back into the nano particles. If toughening of amorphous PET is needed, low loadings of nano Al 2 appear to be good, as the density is not increased greatly. Our main reservation with nano Al 2 was the difficulty in handling it: despite using a respirator mask, it could be felt in the lungs, hence it is hazardous to handle it.

In contrast to Al-amorphous PET, PET composites with graphite, graphene and CNTs show embrittlement at very low addition levels (even at <1 wt %). Alshammary et al.’s [1] work showed that addition of 15% graphite to PET was needed to observe an increase in electrical conductivity; there was a 75% increase in tensile modulus, but the elongation-to-break decreased from 6% in the PET to 1.5% in the composite. Rodríguez-Uicab et al. [4] had a PET with a modulus of 1.33 GPa, a strength of 30.5 MPa, an elongation-to-break of 25%, and a toughness (calculated from the area under the stress-strain curve) of 5.98 J/m^3^. With the addition of just 0.1% multi walled CNTs by melt compounding, Rodríguez-Uicab reported the modulus increased from 1.33 GPa to 1.5 MPa, the strength was 29.7 MPa, but the elongation-to-break decreased from 25% to 2.41%, and the toughness decreased from 5.98 J/m^3^ to 0.47 J/m^3^.; that is, for a very small increase in modulus, a large sacrifice in toughness was made. Aoyama et al. [6] made composites from untreated and treated graphenes in PET. They reported a PET with a tensile modulus of ~1.6 GPa, and an elongation-to-break of ~420%. One of Aoyama’s graphenes at 4% loading gave a small increase in modulus (from 1.6 to 1.8 GPa), with an elongation-to-break of ~90%. Another graphene after surface treatment, at a 2% loading gave a modulus of 2.3 GPa but with an elongation-to-break < 5% [6]. Again, from a mechanical point of view, it translates to a small increase in modulus with a large sacrifice in toughness, made at great expense. Aoyama’s values of modulus and elongation-to-break of the graphene-PET composites [6] may be contrasted with the values in Table 6 and Table 9 for the Al 1-amorphous PET and Al 2-amorphous PET composites, where comparable moduli were obtained but the lowest elongation value was 13.7%; further, Table 7 and Table 10 show all the Al-PET compositions gave increases in the impact resistance over the base PET.

One notable feature is the high discrepancy in elongation-to-break reported for the unfilled PETs. In Alshammary et al., it is 6%, Rodríguez-Uicab et al. cite 25%, but Aoyama et al. [6] had ~420%. We had 96% in Table 6 and Table 9. The reason for this discrepancy could be due to ‘physical ageing’ that takes place in all amorphous polymers [32], and due to the tensile testing rate. Physical ageing leads to a densification and embrittlement with time (which shows also in reduced elongation-to-break). The elongation-to-break of freshly made amorphous PET is ~500% at an extension rate of 2–5 mm/min, but after several months, it decreases. The elongation-to-break also depends on testing rate, with high elongation rates leading to low breakage strains. The physical ageing rate and loss of ductility in amorphous materials depends on storage time and the temperature; the closer the storage temperature is to the *T*_g_, the faster the ageing [29,32]. In our work, we made the measurements within 3 months, and as explained the DSC curves in Figure 8 and Figure 9 did not show the sign of physical ageing at the *T*_g_, hence the amorphous PET bars (with and without Al) can be compared In the other works cited, many do not even specify whether their PET is amorphous or semi-crystalline [1,3,4,5,6] and do not consider the physical ageing phenomenon in amorphous PET, and these have to be deduced indirectly from their DSC curves, or from their sample preparation method. In Rodríguez-Uicab et al.’s [4] work on CNT-PET composites, the DSC suggests the PET in the as-prepared samples was semi-crystalline rather than amorphous, hence the elongation-to-break will be naturally different and cannot be compared with cases where the PET is amorphous. Despite this uncertainty about comparing different works on filled PET, inspection of other polymers with CNTs [33,34] show in most cases, they make the composite brittle even at loadings like 1%.

It is worth having a mental picture of the range of intrinsic impact resistances of unfilled plastics. This range spans the most brittle thermoplastics like amorphous polystyrene with a notched Izod impact resistance of ~12 J/m to the toughest plastic, amorphous polycarbonate, at ~1000 J/m. Polycarbonate with a *T*_g_ of 150°C is an exception, as most thermoplastics with high *T*_g_ fall in the range 25–70 J/m for notched Izod impact resistance. Thermoplastics with low *T*_g_ have higher impact strength; thus, PP homopolymer has a notched Izod impact resistance of ~80 J/m while impact PP copolymers with ethylene comonomer reach up to ~170 J/m, but this is associated with a lowered *T*_g_ and modulus. PET has a higher *T*_g_, and higher modulus and strength than polyolefins. If unnotched, PET has a high impact resistance, but it is a little on the brittle side when the material is notched (notched Izod impact resistance of ~22 J/m). Even with such a value, amorphous PET is a usable material, for example in blood tubes employed in healthcare. For demanding engineering applications, high *T*_g_ polymers are toughened with rubber particles, but this involves a trade-off with sacrificing modulus and strength.

### 3.5. Materials that Show Increase in Modulus and Impact Resistance

The significance of the Al-PET composites has to be weighed against the survey in the Introduction which showed that in metal-polymer composites (and indeed in CNT, graphene and graphite filled PET), the modulus increases, but the impact resistance generally decreases below that of the unfilled plastic, with increase of filler loading [7,8,15,16,17,18,19,20]. Fu et al. [35] reviewed particulate composites with minerals like nano-SiO_2_, glass beads, Al_2_O_3_, Mg(OH)_2_, BaSO_4_ and CaCO_3_ particles. They noted that rigid mineral particles when added to thermosetting resins like epoxies somewhat surprisingly increase the elastic modulus, the toughness, and the hardness [35]. One example was given where Al particles increased the impact toughness of a thermosetting polyester by ~1.5× [35,36]. Yet, with thermoplastics (which are inherently tougher than cross linked thermosets), Fu et al. noted that inclusion of rigid particles usually leads to an increase in modulus but with a significant decrease of fracture toughness [35]. However, there appear to be exceptions with thermoplastics; they cited calcium carbonate filled PP and PE, where large increases in toughness were obtained along with increase in modulus and strength [35,37]. With thermoplastics, the improved toughening by mineral fillers seems to be restricted to the combination of CaCO_3_ with polymers that have sub-ambient *T*_g_s like PE or PP (*T*_g_ of −80 °C and −10 °C respectively) [35,37]. Fu et al. [35] noted that the particle size, the interfacial adhesion and the loading, control the balance of mechanical properties, at least with the mineral particles.

**Figure 11:**
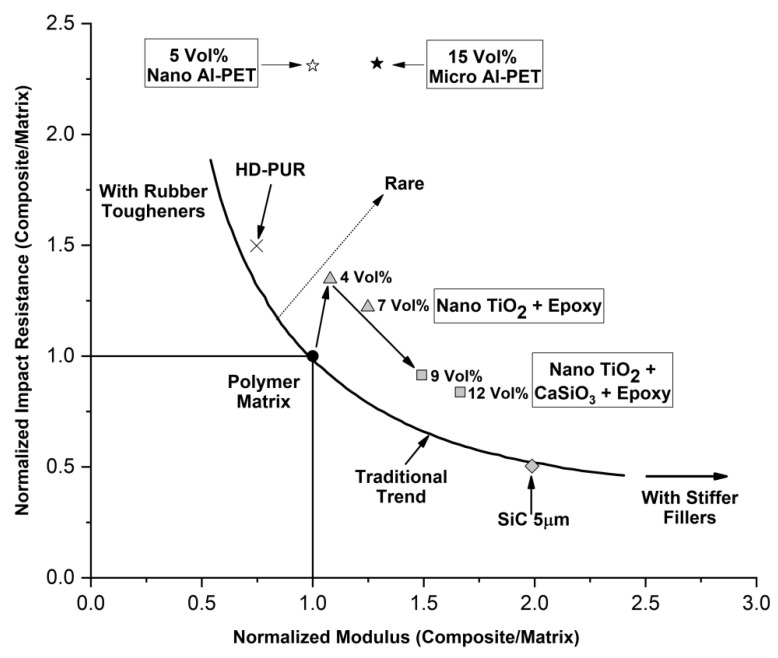
Plot of normalized impact resistance versus normalized modulus, adapted from Wetzel et al. The curve shows the behavior of most filler composites. The dotted arrow shows rare cases where both increase in modulus and impact resistance is obtained. The two points marked with ★ and ✩ are from the Al-PET composites of this work while the others points are adapted from Wetzel et al. [38].

Wetzel et al. [38] studied the incorporation of nano TiO_2_ filler in an epoxy resin to increase its wear resistance. They showed a generalized plot of normalized impact resistance (impact resistance of composite/impact resistance polymer) versus normalized modulus (modulus of composite/modulus polymer) where the curve follows the familiar trend of decreasing impact resistance with increasing modulus. This has been adapted here in Figure 11 and the values of the two Al-composites in this work are placed on the map. The curve passes through (1,1) which corresponds to the normalized values of modulus and impact resistance for the pure PET. In general, rigid fillers like talc, mica, SiC etc., increase the composite modulus (that is normalized modulus > 1), but decrease the impact resistance (normalized impact resistance < 1). On the other hand, polymer fillers with sub-room temperature *T*_g_s give normalized impact resistances > 1 (that is tougher than the polymer), but this leads to the normalized modulus being < 1 (the composite is less stiff than the unfilled polymer); this is observed in the familiar method of rubber toughening of high *T*_g_ plastics. However, Wetzel et al. [38] showed that nano particles of TiO_2_ (300 nm, 4 vol. %) in epoxy achieved a combination of increase in modulus and toughening if the nano TiO_2_ was un-agglomerated. Thus, the normalized modulus was ~1.2 (that is, the composite modulus was 1.2× that of the epoxy) and the normalized impact resistance was 1.3 (impact resistance 1.3× of the epoxy). This is in line with Fu et al.’s review [35] where it was noted that rigid particles increase the impact resistance of thermosets which are inherently brittle.

Adopting a similar approach, from Table 6, the normalized modulus for the 15% Al 1-amorphous PET composite was 2.07 GPa/1.60 GPa = 1.29 and the normalized impact resistance from Table 7 for the 15% A1 1-amorphous PET was (51.56 J/m)/(22.19 J/m) = 2.32, and these were plotted with the ★ in Figure 11. The normalized modulus and impact resistance for the 5 vol. % Al 2-PET composite is plotted with the ✩ using the values from Table 9 and Table 10; in this case, the modulus has not increased (the normalized value is 1), while the impact resistance has more than doubled (2.31). Wetzel’s data [38] on nano TiO_2_ in epoxy are also shown. Instead of improving the impact toughness of amorphous PET with rubber, it is possible to do it with 5 vol. % nano Al 2, without reducing the modulus; however, the density has gone up to 1.401 g/cm^3^ compared with 1.333 g/cm^3^ for amorphous PET.

Thus, Figure 11 demonstrates that both the Al 1-PET and Al 2-PETs show a bigger effect than Wetzel et al.’s nano TiO_2_ in epoxy. In our view, Wetzel’s curve in Figure 11 appears to be correct generally for almost all filler composites. The material space indicated by the dotted arrow in Figure 11, with raised modulus and raised impact resistance, is rare with filler composites, especially with thermoplastic matrices. We have to place Fu and Wang’s CaCO_3_-PE composites [37], and the Al-PET of this work, in the exception list. The Al-PET is a combination where raised modulus and impact resistance were observed with a high *T*_g_ thermoplastic matrix, and this appears to be the first case with a metal-plastic pair.

There is still an unusual feature to reconcile: the extension-to-break of the Al 1 composite dropped drastically at 10 vol. % loading and that of the Al 2 dropped at 5 vol. % loading (see Table 6 and Table 9). Normally, a drop in extension-to-break does correlate with a drop in impact resistance. However, the apparent contradiction in the impact resistance increasing despite the abrupt drop in elongation-to-break can be explained. There is in fact more than one type of impact. There is the familiar Izod impact, but there is also a ‘projectile impact test’ on a flat face, and a ‘tensile impact test’ which is a sudden axial pull along the long axis of the bar. The notched Izod impact test involves a lateral blow in the transverse direction to the bar’s long axis, and the projectile impact on a flat sheet involves sudden compressive stresses. The drop in the tensile extension-to-break implies in a tensile impact test, the Al-PET composites might be brittle (low tensile impact resistance). Thus, it is possible to have a material that has good Izod impact and projectile impact resistance, but which has decreased tensile impact resistance.

The behavior of the Al-PET is a property of this pair. If the Al is replaced with another metal in PET, the same behavior may not occur. Osorio-Ramos, et al. [27] made composites based on recycled polyethylene terephthalate (r-PET) reinforced with Zn metal particles. The preparation method used was not injection moulding; rather, ground recycled-PET (r-PET) powder was mixed with zinc powder, cold pressed together at 350 MPa and sintered at 256 °C for 15 min. The impact resistance increased with the incorporation of Zn from around 8 J/m for neat r-PET to about 11 J/m for composites filled with 40 wt. % Zn particles. Osorio-Ramos’s base value of 8 J/m and the raised value of 11 J/m for the r-PET-Zn composite was lower than for the unfilled amorphous PET here (22.2 J/m) but this maybe because their r-PET had low molecular weight, or possibly due to their preparation method (powder pressing instead of injection moulding), or their PET was crystalline rather than amorphous. In any case, their highest value of 11 J/m for the r-PET-Zn composite, although it represents a substantial increase over their base r-PET, is in absolute terms only half of what we had with amorphous PET (Table 7).

### 3.6. Aluminum-Filled Polycarbonate

**Figure 12:**
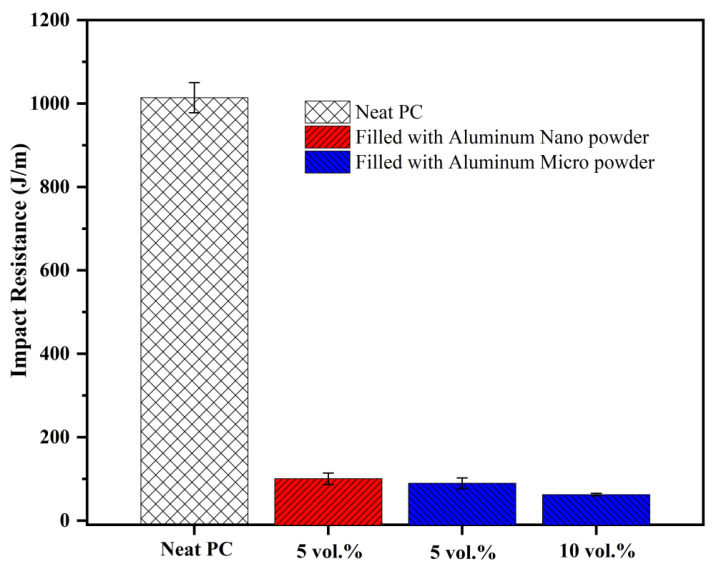
Comparison of Impact resistance of PC and micro and nano Al-PC composites, with standard deviation bars.

Just to check whether our unusual impact result with the Al-PET pair can be replicated with another polymer, we made a composite with polycarbonate (PC) and the same Al micro and nano powders. PC was selected as it is a super tough thermoplastic with a notched Izod impact resistance of 1014 J/m. Figure 12 shows the addition of just 5 vol. % Al 1 or Al 2 powders to PC caused a drastic drop to 90 J/m for Al 1 and 101 J/m for Al 2. Although the reduced value is higher than for Al-toughened PET, PC is a super tough plastic, and this drop in impact would be considered disastrous for this polymer. The Al merely acts as a stress concentrator in the PC.

Some high *T*_g_ polymers such as polystyrene and PMMA are prone to crazing and the material fails by this mode. Such polymers have higher yield strengths than the breaking strengths. These polymers have both low crack initiation and low propagation energies; hence, they show low un-notched and notched Izod impact resistances. Some polymers like polyamides, polycarbonate and PET tend to fail by shear yielding. This mode has high crack initiation energy but low crack propagation energy [39]. Tanrattanakul et al. stated that un-notched PET is tough, but notching reduces the impact resistance [40]. As a final word on the impact toughening, the mechanisms of rubber toughening are complex and are not completely resolved, but there is a large body of information on it, and it is widely used commercially for toughening engineering thermoplastics, although it will always mean some sacrifice in modulus. [39]. ‘Rigid-rigid’ toughening of thermoplastics is also a known method whereby a high *T*_g_ thermoplastic like poly(phenylene oxide) is added to polypropylene, to form a particulate composite [41]. However, the principles by which metal particles could toughen a thermoplastic are not known—indeed, we believe this is a first report of this kind.

We think that Al is an unusual material as a filler for the following reasons. Pure aluminum’s modulus is 70 GPa whereas other metals, inorganic ceramics and CNTS have modulus well over 100 GPa (CNTs have axis modulus in the range of 700–1000 GPa). Al’s extension-to-break is high at 50–70%, while the ceramics and CNTs have extension-to-break in the range of 1 or 2%. E-glass has a modulus of 72 GPa (similar to Al) but its extension-to-break is low (4.8%). Alumina (Al_2_O_3_) has a higher modulus than glass of 330–435 GPa but its extension-to-break is even lower (0.7%). Rubbers have high extensibility {>100%) but their modulus is very low (1–5 MPa), thus their addition leads to toughening but a decrease of the composite’s modulus. But a material like pure Al because of its intermediate modulus of 70 GPa with extension of 50–70%, if it bonds well to the polymer, will increase the modulus and impact resistance. In the case of PET, the bonding with Al is natural, but with other composites, the polymers or Al may have to be treated to give good bonding, and then the same properties observed with amorphous PET and Al may be attainable.

### 3.7. Thermal Conductivity

The thermal conductivity values for the amorphous PET and the Al-PET composites are shown in Table 12. A gradual increase in the thermal conductivity of the composites was observed with increase in loading of both the nano as well as the micro aluminum particles. This is similar to other metal-plastic composites. Unlike electrical conductivity, generally the threshold where there is an up-shoot in thermal conductivity is near 70% loading. However, no upshoot in the thermal conductivity was observed with the two Al fillers. In both cases, we were below the threshold concentration where the thermal conductivity shoots up. In the 20 vol. % Al 2-amorphous PET composite, the thermal conductivity was over 2× the base material.

There is demand for plastic materials with high thermal conductivity but without high electrical conductivity. Such materials are sought for heat dissipation in miniaturised electronics, and also due to the present drive for electrification of cars. Commercial, thermally-conductive composites achieve a through-the-plane thermal conductivity of 0.5 to 2 W/m K; most often, these are composites with glass fibre included (besides the thermally conductive additive) to raise the modulus to ~10–13 GPa. The 20% Al 1-PET composite with a thermal conductivity of 0.5034 W/m K attained the bottom end of the commercial range, but the advantage is the impact resistance was not compromised. Most commonly in commercial formulations with thermoplastics, alumina and boron nitride are used for the thermally conductive filler, but high loadings are needed (>50%, near 70%), and at such high levels, the composite would be brittle.

Graphene and CNTs can be used to increase thermal conductivity, but besides their high expense, they would increase electrical conductivity. Therefore ‘hybrid filler’ composites are being researched. Akthar et al. [7] described hybrid fillers in which the thermally conductive component that normally needs a high loading if acting on its own was joined with an expensive filler with extremely high intrinsic thermal conductivity. In their work [7], alumina-graphene hybrid fillers were synthesized and then added to epoxy. Both fillers were modified with silanes to develop the interface bonds with the epoxy. The thermal conductivity of the neat epoxy was 0.2 W/m K which is similar to the PET (Table 12). Using a 50 vol. % alumina-1 wt.% graphene hybrid filler in a particular epoxy, the composite attained a thermal conductivity of 1.681 W/m K. However, the problem with the system is the cost of the graphene, the procedure of making the hybrid with the inorganic alumina [7], and the surface treatments. Although the epoxy system is used as a ‘thermal interface material’, which bridges a heat source like a semi-conductor chip to a heat sink, it is not an injection mouldable composition for complex shapes. Akthar et al. [7] did not measure the mechanical properties, but almost certainly their hybrid filler-epoxy composite would be brittle, although as a bridging interface, that would not matter. With the Al-PET, no surface treatment is needed for adhesion of the filler with the polymer, it is easily injection mouldable, and the material is in fact toughened.

In this work, the PET was in an amorphous state after injection moulding, in which case the end-use temperature for any Al-PET composites made thus has to below the *T*_g_, that is 78 °C. If the PET could be in a crystalline state, then the deformation temperature would be controlled by its *T*_m_ which is ~250 °C. To making crystalline PET mouldings, hot moulds would be needed, as PET is a slow crystallizer, despite the nucleating effect of the Al particles. In the current work, hot moulds were not available. Attempts were made to post-crystallize the Al-amorphous PET bars in an oven at 170 °C. However, this led to shrinkage and warping of the bars, hence it was inappropriate to use them for tensile tests and impact tests, to see if the results extend to when the PET is in a crystallized state. Thus, at this stage we do not know whether the unusual combination of increased tensile modulus and strength, with increased impact resistance will be observed if the PET matrix was crystallized. The crystallization of the bars has to be done in the mould itself.

## 4. Conclusions

Amorphous PET composites containing micro and nominally nano aluminum powders were made after the observation that PET melt in contact with aluminum foil bonds strongly to it. As with other particle-filled composites, the modulus showed a modest increase. In almost all cases of filler composites with thermoplastic matrices, the tensile strength and notched Izod impact resistance decrease below the base polymer. This applies to most combination of polymers with metal particles, inorganic particles, CNTs and graphene. However with Al-PET, the tensile strength did not drop while the notched Izod impact resistance more than doubled at 15 vol. % micron-sized Al. The nano Al 2 particles showed agglomerates which were tens of microns in size, yet composites made from them also showed an increase in impact resistance, with no drop in modulus and strength; at 5 vol. %, the notched Izod impact resistance more than doubled with the nano Al 2 as well, and the standard deviation was low. Thus, the remarkable feature of the Al-PET composite was that it was a material where the modulus increased, the strength did not drop, and the impact resistance increased at all compositions (there was never a decrease below the base amorphous PET). This is a rare combination.

SEM pictures showed the Al particles generally bonded well to the PET with not many pull outs. Aluminum generally has an oxide coating and this was not removed, nor were any other coatings applied. The increase in modulus and impact resistance is peculiar to this metal-plastic pair. Tests with the same Al powders with polycarbonate showed a drastic drop in impact resistance at any loading of Al.

Generally, the inapplicability of metal-plastic, CNT-plastic and graphene-plastic composites is because by the concentration where the percolation threshold is reached, the mechanical properties decrease to such an extent that the material is unusable. In most of the literature, at the metal loadings needed to get high electrical conductivity, the composite becomes unacceptably weak and brittle (low tensile strength and brittle). Here, we did not have the equipment to measure the electrical conductivity, but we believe that by adjusting the metal content and particle shape, an Al loading will be found where raised electrical conductivity will be obtained with adequate impact resistance. The thermal conductivity showed a 2× increase at 20 vol. % of micro Al 1. Clearly, we were still below the percolation threshold for thermal conductivity.

The Al 2-PET composites with loadings up to 3 vol. % retained the high extensibility (5–6×) of PET above the *T*_g_. This would allow oriented, conductive fibres to be made. For this application, it is important to have fine nano size Al without agglomeration.

The PET in the composite mouldings was amorphous as heated moulds were not available. In many recent works using PET as the matrix for CNT and graphene composites, many authors do not take care to describe whether the PET is amorphous or semi-crystalline. We recommend that this is carefully established as in amorphous PET, embrittlement occurs with time and temperature, due to physical ageing. The easiest way to detect physical ageing is the appearance of a hook or peak in the DSC at the *T*_g_. The Al particles acted as a nucleating agent, as the cold crystallization peak shifted to lower temperatures on heating above the *T*_g_, while on cooling from the melt, crystallization started earlier than normal. However, the aluminum did not increase the overall crystallisation speed to such an extent as that of the injection mouldable polyester PBT (the fact the PET phase in the Al-PET bars moulded with cold moulds were amorphous testifies to this). Room temperature applications of Al-filled amorphous PET are possible if the service temperature is limited to below the *T*_g_ (78 °C).

Further work will look at (1) the effect of Al particle shape (2) crystallized PET matrix and (3) electrical and thermal conductivity, if possible, at lower loadings. We think that it is worth exploring CNT/Al and graphene/Al hybrids, to see if the conductive properties with toughness can be obtained by a combination of both. To be successful as a conductive plastic, the combination of materials should show the right balance of conductivity, mechanical properties, ease of processing, and price.

## Figures and Tables

**Table 1:** Physical properties according to the data sheets of the Aluminum micro and nano powders from Nanokar, Turkey.

Property	Micro Al 1 Powder	Nano Al 2 Powder
Particle size	3 µm	40–60 nm
Purity	≥99.7%	≥99%
Appearance	Grey powder	Grey powder
Morphology	Nearly spherical	Spherical
Density	2.7 g/cm^3^	2.7 g/cm^3^

**Table 2:** Aluminum loading percentages and theoretical densities of Micro-Al 1/amorphous PET composites. Note that for 20% and 30% Al 1, tensile bars could not be moulded without defects. However, Izod bars could be moulded for all compositions.

Sample ID	Al Loading (vol. %)	Corresponding Al Loading (wt. %)	Theoretical Sample Density (g/cm^3^)
0% Al/PET	0	0.00	1.333
5% Al/PET	5	9.63	1.401
10% Al/PET	10	18.37	1.469
15% Al/PET	15	26.33	1.538
20% Al/PET	20	33.62	1.606
30% Al/PET	30	46.47	1.743

**Table 3:** Aluminum loading percentages and theoretical densities of Nano Al 2/amorphous PET composites.

Sample ID	Al Loading (vol. %)	Corresponding Al Loading (wt. %)	Theoretical Sample Density (g/cm^3^)
0% Al/PET	0	0.00	1.333
1% Al/PET	1	2.00	1.347
3% Al/PET	3	5.90	1.374
5% Al/PET	5	9.63	1.401

**Table 4:** Thermal parameters obtained from the first cycle of DSC thermographs of neat amorphous PET and micro Al 1/amorphous PET composites (Figure 8). *T*_g_ is the glass transition; *T*_m_ is the melting peak temperature; *T*_cc_ is cold crystallisation peak temperature from the amorphous state and *T*_c_ is peak temperature for crystallisation from the melt.

1st Cycle							
Sample	*T*_g_	*T*_cc_	∆*H*_cc_	*T*_m_	∆*H*_m_	*T*_c_	∆*H*_c_
	°C	°C	J/g	°C	J/g	°C	J/g
Neat PET	78.1	142.6	−27.7	249.1	40.80	186.1	−38.0
5 vol. % Al 1/PET	77.5	136.0	−25.9	248.6	42.67	196.5	−40.6
10 vol. % Al 1/PET	77.5	134.1	−25.3	249.6	39.65	197.9	−36.1
15 vol. % Al 1/PET	77.5	139.5	−23.7	249.9	42.88	195.8	−32.5
20 vol. % Al 1/PET	77.5	130.3	−18.6	251.0	40.84	208.7	−29.4

**Table 5:** Thermal parameters obtained from the first cycle of DSC thermographs of neat amorphous PET and nano Al 2/amorphous PET composites.

First Cycle							
Sample	*T*_g_	*T*_cc_	∆*H*_cc_	*T*_m_	∆H_m_	*T*_c_	∆*H*_c_
	°C	°C	J/g	°C	J/g	°C	J/g
Neat PET	78.1	142.6	−27.7	249.1	40.8	186.1	−38.0
1% Al 2/PET	76.3	137.9	−26.4	248.9	41.21	198.0	−42.8
3%Al 2/PET	76.3	140.0	−25.0	248.4	36.70	192.6	−34.4
5% Al 2/PET	76.3	139.0	−24.7	248.9	40.21	194.4	−37.0

**Table 6:** Tensile properties of amorphous PET amorphous PET and Al 1/amorphous PET composites. Std. Dev. is the standard deviation.

Sample	Tensile Modulus (GPa)	Std. Dev.	Tensile Strength (MPa)	St. Dev.	Elongation at Break (%)	Std. Dev.	Flexural Modulus (GPa)	Std. Dev.	Flexural Strength (MPa)	Std. Dev.
Neat PET	1.60	0.03	59.9	0.60	96.2	71.7	2.47	0.10	89.0	3.4
5% vol.	1.75	0.05	56.6	0.61	135.7	158.0	2.74	0.04	88.6	0.5
10% vol.	1.95	0.08	58.3	1.43	16.1	8.0	3.18	0.10	92.8	2.7
15% vol.	2.07	0.09	57.2	0.72	13.7	2.4	3.24	0.23	90.5	1.5

**Table 7:** Notched Izod Impact resistance in J/m of amorphous PET and Al 1/amorphous PET composites. Std. Dev. is the standard deviation. N is number of tests.

Sample	N	Mean Izod Impact Value (J/m)	Std. Dev.	Minimum	Median	Maximum	Range
Neat PET	10	22.19	6.66	12.50	21.88	37.50	25.00
5 vol. %	10	30.31	9.09	21.88	29.69	50.00	28.13
10 vol. %	10	38.75	7.54	28.13	39.06	50.00	21.88
15 vol. %	10	51.56	8.24	31.25	56.25	56.25	25.00
20 vol. %	10	41.88	12.77	25.00	40.63	68.75	43.75
30 vol. %	10	40.63	7.06	28.13	39.06	50.00	21.88

**Table 8:** For the unpaired *t*-test, the null hypothesis was there is no difference between the means of the two compositions that are compared, and the alternative hypothesis applied was the two means are different at the 5% significance level. ‘Significant’ means the alternative hypothesis is accepted and there is a difference between the means, and ‘non-significant’ means we accept the null hypothesis that there is no difference in the means. vs. is ‘versus’.

*T*-Test Comparison of Means of Izod Impact, for Two Compositions	*T*-Test Significance at 5% Level, for Difference of Means of Izod Impact Resistance
Any Al 1 composition vs. Neat Amor. PET	Significantly Different
5 vol. % Al 1 vs. Any other Al 1 composition	Significantly Different
10 vol. % vs. 15 vol. % Al 1	Significantly Different
10 vol. % vs. 20 vol. % Al 1	Not significantly Different
10 vol. % vs. 30 vol. % Al 1	Not significantly Different
15 vol. % vs. 20 vol. % Al 1	Not significantly Different
15 vol. % vs. 30 vol. % Al 1	Significantly Different
20 vol. % vs. 30 vol. % Al 1	Not significantly Different

**Table 9:** Tensile Properties of Al 2/amorphous PET composites. Std. Dev. is the standard deviation.

Sample	Tensile Modulus (GPa)	Std. Dev.	Tensile Strength (MPa)	St. Dev.	Elongation at Break (%)	Std. Dev.	Flexural Modulus (GPa)	Std. Dev.	Flexural Strength (MPa)	Std. Dev.
Neat PET	1.60	0.03	59.9	0.60	96.2	71.7	2.47	0.10	89.0	3.4
1 vol. %	1.54	0.04	55.6	0.29	442.1	7.2	2.52	0.03	89.4	0.5
3 vol. %	1.63	0.03	56.2	0.78	428.3	8.8	2.65	0.03	90.1	0.1
5 vol. %	1.61	0.05	56.0	0.44	201.9	148.3	2.78	0.02	90.3	0.2

**Table 10:** Notched Izod Impact resistance in J/m, of amorphous PET and Al 2/Amorphous PET composites. Std. Dev. is the standard deviation. N is the number of tests.

Sample	N	Mean Izod Impact Value (J/m)	Std. Dev.	Minimum	Median	Maximum	Range
Neat PET	10	22.19	6.66	12.50	21.88	37.50	25.00
1 vol. %	10	35.00	10.29	21.88	31.25	50.00	28.13
3 vol. %	10	42.19	8.10	25.00	46.88	50.00	25.00
5 vol. %	10	51.25	3.36	46.88	50.00	59.38	12.50

**Table 11:** Unpaired *t*-test for Al 2—PET compositions to determine in the means of Izod impact values are different at the 5% significance level. The same null and alternative hypotheses were used as in Table 8. vs. is versus.

*T*-Test Comparison of Means of Izod Impact, for Two Compositions	*T*-Test Significance at 5% Level, for Difference of Means of Izod Impact Resistance
Any Al 2 composition vs. Neat Amor. PET	Significantly Different
1 vol. % vs. 3 vol. % Al 2	Not significantly Different
1 vol. % vs. 5 vol. % Al 2	Significantly Different
3 vol. % vs. 5 vol. % Al 2	Significantly Different

**Table 12:** Thermal Conductivity of amorphous PET and nano-Al/amorphous PET composites.

Sample	Thermal Conductivity (W/m K)
Amorphous PET	0.2434
PET-Micro Al 1 (5 vol. %)	0.2865
PET-Micro Al 1 (10 vol. %)	0.3385
PET-Micro Al 1 (15 vol. %)	0.3929
PET-Micro Al 1 (20 vol. %)	0.5034
PET-Nano Al 2 (1 vol. %)	0.2462
PET-Nano Al 2 (3 vol. %)	0.2695
PET-Nano Al 2 (5 vol. %)	0.2933
